# Osteosarcopenia in Very Old Age Adults After Hip Fracture: A Real-World Therapeutic Standpoint

**DOI:** 10.3389/fmed.2021.612506

**Published:** 2021-05-20

**Authors:** Monica Pizzonia, Andrea Casabella, Marta Natali, Lorena Petrocchi, Luca Carmisciano, Alessio Nencioni, Luigi Molfetta, Chiara Giannotti, Gerolamo Bianchi, Andrea Giusti, Federico Santolini, Fiammetta Monacelli

**Affiliations:** ^1^Istituto di Ricovero e Cura a Carattere Scientifico Ospedale Policlinico San Martino, Genoa, Italy; ^2^DIMI, Department of Internal Medicine and Medical Specialties, Section of Geriatrics, University of Genoa, Genoa, Italy; ^3^DISSAL, Department of Health Science, University of Genoa, Genoa, Italy; ^4^DISC, Department of Integrated Surgical and Diagnostic Sciences, University of Genoa, Genoa, Italy; ^5^Rheumatology Unit, Department of Musculoskeletal Sciences, Local Health Trust 3, La Colletta Hospital, Genoa, Italy

**Keywords:** longitudinal assessment, muscle strength, denosumab, osteosarcopenia, hip fractured very old age patients

## Abstract

Loss of bone and muscle mass and strength (i. e., osteosarcopenia) is a highly prevalent clinical condition in older adults, associated with an increased risk of fragility fractures and unfavorable clinical outcomes. Although sarcopenia is a potential risk factor for osteoporosis and subsequent fracture, and the management of this hazardous duet is the key to preventing osteoporotic fracture, evidence pertaining to the treatment of sarcopenia for the purpose of preventing fragile fractures remains insufficient. Given this scenario we aimed at prospectively compare the long-term effectiveness of bisphosphonates vs. denosumab, on bone and muscle, in a cohort of old age hip fractured patients by virtue of a timely osteo-metabolic and sarcopenic assessment. Ninety-eight patients consecutively enrolled at the IRCCS Hospital San martino, Genoa, Italy, received at baseline comprehensive geriatric assessment and Bone Densitometry (DXA) with the quantitative and quantitative bone analysis and evaluation of relative skeletal muscle index (RSMI) and longitudinally after 1 year form hip surgery. The results showed a slightly and non-significant osteo-metabolic improvement in the Alendronate group compared to the Denosumab group, and a positive trend of RSMI measurements in the Denosumab group. Although preliminary in nature, this is the first report to longitudinally analyze osteosarcopenia in a real-world cohort of very old age patients after hip fracture and moved a step forward in the understanding of the best osteo-metabolic therapy for long- term treatment, exploring as well the potential dual role of denousumab as antiresorptive and muscle strength specific drug for osteosarcopenia in this vulnerable population.

## Introduction

It is growingly acknowledged that the increase of fracture risk with aging reflects a multifactorial basis, including bone mineral density (BMD) loss, poorer bone quality with insufficient bone strength, and overarching the diagnosis of osteoporosis. In line with that, it is generally accepted that osteoporosis (1, 2) is a major clinical problem in older adults that can have a significant impact on the day life activities, and similarly, substantial long-term morbidity is associated with hip fractures. Namely, the disability, mortality, and cost of hip and vertebral fractures, the most burdening osteoporosis-related complications, are substantial in the rapidly growing aging population so that prevention and treatment of osteoporosis is a major public health concern (3).

However, from a clinical standpoint, it is increasingly recognized that the simultaneous presence of bone and muscle weakness dramatically contributes to higher fracture risk in older adults, and this is especially true in presence of clinical frailty, that is, a very common geriatric syndrome characterized by diminished homeostasis and increased susceptibility to environmental stressors with increased unfavorable clinical outcomes.

The term *sarcopenia*, describes an “age-associated loss of skeletal muscle mass and functions which are strength and performance as well” (4). Doubtlessly, it is a widespread clinical condition of the old age, that is tightly associated with key relevant clinical complications, such as functional decline, physical disability, mobility limitations, increased risk of falls, and poorer quality of life (5). Sarcopenia has been reported to affect more than 40% of older adults ≥70 years of age, ~50 million people worldwide. This number is estimated to increase to 500 million people in the year 2050 (6, 7), and although it is frequently appreciated by clinicians, it is rarely formally diagnosed.

As reported by the milestone paper of Brinkley et al., the time has come to emphasize the key relevance of this duality, suggesting that the definition of sarco-osteoporosis is proposed to facilitate the early identification and appropriate clinical management of those old-age patients at higher fracture risk (1).

Some evidence showed that sarcopenia is associated with decreased bone density, and common risk factors such as vitamin D deficiency, malnutrition, and disuse have been reported to lead simultaneously to loss of bone and bone strength with decreased muscle mass and a higher predisposition to falls (8–10), suggesting that bone fractures, including both hip and vertebral fractures, are caused by a combination of osteoporosis and sarcopenia. Additionally, osteosarcopenia has been related to the development of dysmotility syndromes that are a key relevant complication for old-age patients, accelerating disability and frailty trajectories (11, 12).

Notwithstanding that, there has been a delay in the understanding of the pathophysiology of muscular regeneration and sarcopenia in the aged environment (13) that could be responsible, at least partially, for the limited awareness pertaining the cooccurrence of sarcopenia in patients with osteoporotic (OP) fractures in old-aged populations (12–16). As a result, a fragmentation in the timely diagnosis and in the appropriateness of treatments has been observed, especially in multimorbid, frail old-age patients. Although sarcopenia is a potential risk factor for osteoporosis and subsequent fracture, and the management of this *hazardous duet* is the key to preventing OP fracture, evidence pertaining to the treatment of sarcopenia for the purpose of preventing fragile fractures remains insufficient (17–22).

Whereas, several drugs are approved for the treatment of osteoporosis, so far, no therapy has been demonstrated to exert sufficiently positive effects on muscle to be approved for the treatment of sarcopenia. It is well-known that a monoclonal antibody targeting RANKL, denosumab, was observed to reduce fracture risk, and it is now widely used to treat osteoporosis (23). RANKL is also expressed in skeletal muscle and activation of the NF-κB pathway mainly inhibits myogenic differentiation, which leads to skeletal muscle dysfunction and loss (24). In line with that, limited *in vivo* evidence underscored that a neutralizing antibody against receptor activator of the NF-kB ligand (RANKL), denosumab, improved muscle strength and insulin sensitivity, restoring bone strength. In addition, OP women, taking denosumab for more than 3 years, ameliorated their appendicular lean mass and hand-grip strength compared with no treatment, whereas the use of bisphosphonate did not (25). These observations led to hypothesize that RANKL inhibitors could exert a positive influence on muscle mass and strength, particularly in conditions of osteoporosis and/or sarcopenia.

Given this scenario, the aim of the study was to prospectively compare the long-term effectiveness of bisphosphonates vs. denosumab, on bone and muscle, in a cohort of old-age hip fractured patients by virtue of a timely osteo-metabolic and sarcopenic assessment.

## Subjects and Methods

**Figure 1:**
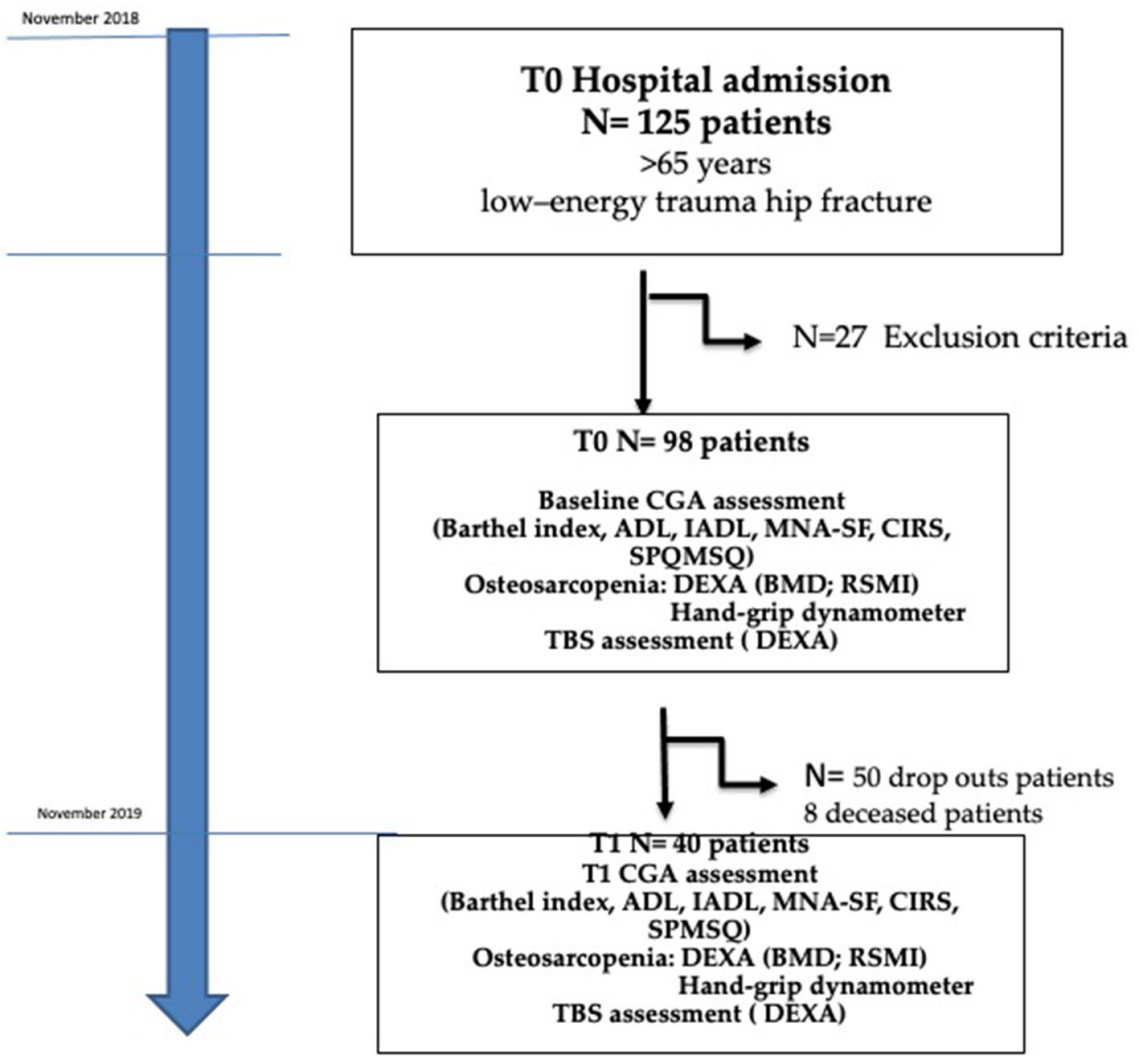
Patients' flowchart assignment.

We performed an observational prospective study, including 125 consecutive old-age patients with hip fracture admitted at the U.O. Emergency Orthopedics and Traumatology of the IRCSS Policlinico San Martino hospital Genoa, Italy, with geriatric co-management, between April and November 2018. The protocol was approved by the Local Ethical Committee and met guidelines of the local governmental agency. Patients or their proxies provided written informed consent prior to study inclusion. Qualified patients were ≥65 years old and had sustained hip fractures due to low-energy trauma (fragility fractures), requiring immediate hospitalization. They were excluded if informed consent was lacking, surgery was prohibited by surgical or clinical instability, high-energy trauma was involved, or fractures were pathologic or periprosthetic in nature. Twenty-seven patients met exclusion criteria for clinical instability (*n* = 14) and pathological fractures (*n* = 13) leaving a total of 98 patients for study (Figure 1).

All patients received in-hospital comprehensive geriatric assessment (CGA), including the Barthel index (26), to assess functional status; activities of daily living (ADL) to assess functional status (27); instrumental activities of daily living (IADL) to assess instrumental activities of daily living (28); cumulative illness rating scale (CIRS) to assess multimorbidity (29); mini nutritional assessment (MNA-SF) (30); and short portable mental status questionnaire (SPMSQ) to assess cognitive status (31, 32).

The diagnosis of sarco-osteoporosis was formulated in the presence of the combination of Dual Energy X-ray Absorptiometry (DEXA) values for poor BMD, the clinical presence of hip fracture, and the cut-off values for poor relative skeletal muscle index (RSMI) as above described. The inclusion of hand-grip strength with cut off was also included for the diagnosis of sarcopenia (4).

Bone densitometry (DXA Lunar Prodigy Scan Advance Ge Medical) with the quantitative analysis of the BMD analysis was performed to assess osteoporosis. Namely, the expressed in g/cm^2^ at the lumbar spine (L1–L4) and femoral neck along with the whole body and seven different anatomic areas (head, upper limbs, lower limbs, trunk, spine, ribs, pelvis). Subjects were classified as osteopenic (T-score = −1.0 to −2.4 DS) or OP (T-score < −2.5 DS) according to the T-score.

The bone qualitative assessment was also investigated with the trabecular bone score (TBS) analysis [iNsight (Medimaps group/GE Healthcare Needham, MA, USA, software version 2.1.0.0)]. The TBS is an index of bone quality derived from dual-energy (33). The lumbar spine L1–L4 TBS (unit-less) was calculated on each spine DXA examination. A normal range for TBS values in postmenopausal women has been proposed as follows: a TBS of ≥1.350 is considered normal; a TBS between 1.200 and 1.350 is considered consistent with partially degraded microarchitecture, and a TBS of ≤1.200 defines degraded microarchitecture (34).

A dedicated software analyzed, by non-invasive techniques, the whole-body composition and the different body composition of three major areas (arms, legs, and trunk) describing total body mass (TM) (gr), total lean mass (LM) (gr), total fat mass (FM) (gr) and bone mineral content (BMC) (gr) for each area. The RSMI was calculated using Baumgartner's equation and, according to the European Working Group on Sarcopenia in Older People criteria (4), we classified patients by having or not sarcopenia; RSMI was derived from the ratio between appendicular skeletal lean mass and height squared and sarcopenia is defined with values (< 5.5 kg/m^2^ in women and < 7.26 kg/m^2^ in men). The limited radiogenic emission required for the study of body composition has allowed the use of this method without invasiveness indices. The limited radiogenic emission required for the study of body composition has allowed the use of this method without invasiveness indices.

All patients performed Hand-Grip assessment to screen for sarcopenia (Camry; EH101 Units: Kg/libbers; Maximum capacity 90 Kg; Power 2X 1.5 V AAA batteries; Tolerance ± 0.5 Kg dynamometer) and lower cut-off values for sarcopenia were defined, respectively, as handgrip measurement <27 kg for males and <16 kg for females (35).

Patients were assigned to alendronate and/or denosumab anti-OP regimens, according to patient's clinical characteristics and based on the AIFA 79 prescription note (36). Namely, 26 patients were assigned to bisphosphonate treatment with alendronate 70 mg once a week, and 15 patients were assigned to denosumab treatment 60 mg 1 fl *via* subcutaneous injection one every 6 months, respectively, due to the presence of renal failure (clearance < 30 ml/min) (70%) for the presence of esophagitis (5%) and the inability to comply with alendronate administration regimen (25%).

Data were collected at baseline (T0) and prospectively after 1 year (T1) from hip surgery at the orthogeriatric outpatient's office of the same hospital. This was an intention to treat analysis irrespective of patient's compliance and adherence to the prescribed drug regimens.

## Statistical Analysis

Continuous variables were described as mean and standard deviation (SD) or median and inter-quartile range (IQR) and compared, respectively, with *t*-test or Mann-Whitney test. Categorical variables were expressed as number and proportion of patients and compared using the Chi-squared test.

The improvement of osteosarcopenic parameters was defined as any positive value of the difference between the last (after 1 year) and the first assessment. Unavailable measurements at the follow-up time were considered as not improved.

*P*-value < 0.05 was considered statistically significant. *P*-value < 0.005 was considered statistically significant. R-software version 4.0.2 (37) was used for all statistical analyses.

## Results

### Baseline Clinical and Osteo-Metabolic Characteristics

Patients' clinical characteristics along with mean DEXA osteosarcopenic parameters, including median BMD scores, TBS scores, and RSMI scores are summarized in Table 1. Mean patients' ages were 82.1 years (SD = 5.8, range 67–94), and women were 87.5%. Baseline characteristics were comparable between treatment groups (Table 1), except for a lower BMI that was observed in the denosumab group compared to the alendronate group (median difference = −2.4, Mann-Whitney *p* = 0.069) and a slightly higher baseline TBS value in the denosumab group compared to the alendronate group (median difference = +0.08, Mann-Whitney *p* = 0.050).

**Table 1:** Clinical characteristics of the study population along with mean DEXA osteo-metabolic parameters.

	**Overall, *N* = 40**	**Alendronate, *N* = 25**	**Denosumab, *N* = 15**	***p***
Age, Mean (SD)	82.1 (5.8)	81.4 (6.3)	83.3 (4.8)	0.315
Female, *N* (%)	35 (87.5)	22 (88.0)	13 (86.7)	0.999
Hip fracture type	16 (40.0)	12 (48.0)	4 (26.7)	0.317
BMI, Median (IQR)	22.7 (20.5, 25.6)	23.4 (20.6, 27.9)	21.0 (19.4, 23.6)	0.069
**Hand Grip, Median (IQR)**				
Females	15.70 (10.85, 17.75)	15.75 (11.10, 17.47)	15.00 (10.00, 17.80)	0.785
Males	22.50 (22.10, 24.30)	22.50 (19.70, 24.70)	23.20 (22.65, 23.75)	0.999
Barthel, Median (IQR)	90 (75, 100)	90 (70, 95)	95 (85, 100)	0.143
ADL, Median (IQR)	6 (5, 6)	6 (6, 6)	6 (5, 6)	0.247
IADL, Median (IQR)	7.0 (3.8, 8.0)	7.0 (5.0, 8.0)	7.0 (1.5, 8.0)	0.597
MNA-SF, Median (IQR)	12 (9, 13)	12 (10, 13)	12 (9, 12.5)	0.357
CIRS comorbidity, Median (IQR)	4.0 (2.8, 5.2)	4.0 (2.0, 5.0)	4.0 (3.5, 5.5)	0.977
CIRS severity, Median (IQR)	1.8 (1.6, 2.1)	1.8 (1.5, 2.1)	1.9 (1.7, 2.1)	0.769
N. of drugs, Median (IQR)	5.0 (3.0, 7.0)	5.0 (2.0, 7.0)	5.0 (4.5, 8.0)	0.338
<5	15 (37.5)	11 (44.0)	4 (26.7)	0.367
5–7	16 (40.0)	10 (40.0)	6 (40.0)	
8 or more	9 (22.5)	4 (16.0)	5 (33.3)	
SPMSQ Median (IQR)	1.0 (0.0, 3.0)	1.0 (0.0, 3.0)	2.0 (0.0, 3.0)	0.954
VIT.D 25-OH (ng/ml), Median (IQR)	15.60 (7.53, 25.45)	15.40 (6.20, 22.00)	21.40 (9.45, 26.60)	0.295
PTH (ng/L) Median (IQR)	33.50 (25.00, 50.50)	33.00 (25.00, 48.00)	35.00 (26.50, 51.50)	0.557
FN-BMD, Median (IQR)	0.65 (0.62, 0.73)	0.66 (0.63, 0.72)	0.63 (0.59, 0.73)	0.395
TH-BMD, Median (IQR)	0.72 (0.65, 0.77)	0.72 (0.65, 0.77)	0.72 (0.63, 0.78)	0.893
LS-BMD, Median (IQR)	0.96 (0.87, 1.07)	0.97 (0.88, 1.14)	0.91 (0.82, 1.03)	0.235
TBS, Median (IQR)	1.09 (0.96, 1.18)	1.07 (0.92, 1.13)	1.15 (1.06, 1.23)	0.050
RSMI kg/m^2^, Median (IQR)	5.90 (5.12, 6.86)	6.22 (5.18, 7.02)	5.76 (5.08, 6.60)	0.299

*BMI, body mass index; FN, Femoral neck; LS, Lumbar spine; TH, Total hip; ADL, activities of daily living; IADL, instrumental activities of daily living; CIRS, cumulative illness rating scale; MNA-SF, Mini nutritional assessment; SPMQS, short portable mental status questionnaire; PHT, parathormone; BMD, Bone marrow density; TBS, Trabecular bone score; RSMI, Relative skeletal muscle index*.

### The Longitudinal Patients' Osteosarcopenic Assessment After 1-Year From Hospital Dischage

Forty patients out of the 98 patients enrolled at baseline underwent longitudinal assessment (T1) after 1-year from hospital discharge and were included in the final analysis. Namely, eight patients deceased, and 50 patients discontinued the follow-up for higher istituzionalization rate, accounting for the higher dropout rate at follow-up.

**Figure 2:**
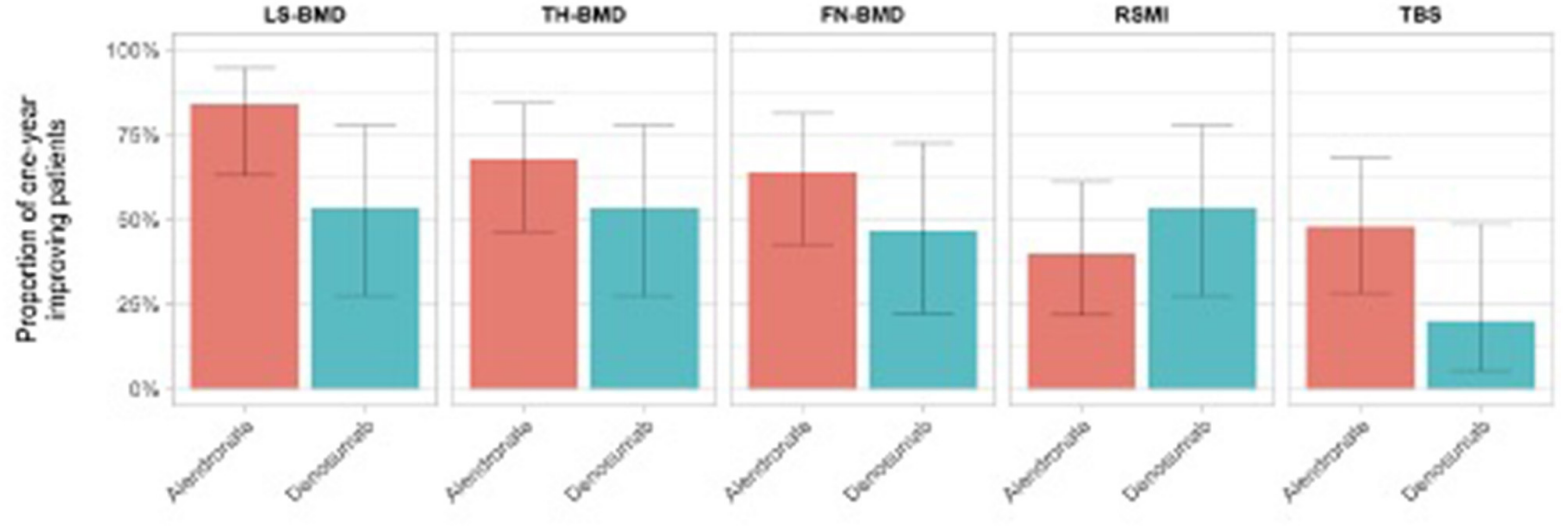
Proportion of patients with osteosarcopenic improvement at T1 assessment on the basis of the assigned treatment group.

The number of treatment responders for each osteosarcopenic parameters is reported in Table 2. At T1 assessment, we observed a slightly and non-significant BMD improvement in the alendronate group compared to the denosumab group, in all measured BMD districts (FN: 64.0 vs. 46.7%; TH: 68.0 vs. 53.3%; LS: 84.0 vs. 53.3%). Similarly, a slight and non-significant higher proportion of patients in the alendronate group showed a mild improvement of bone quality (TBS scores) compared to the denosumab group (48.0 vs. 20%), respectively. Moreover, a positive trend of RSMI measurements was observed in the denosumab group compared to the alendronate group (53.3 vs. 40%) (Figure 2).

**Table 2:** Proportion of patients with osteo-metabolic and sarcopenic improvement at T1 assessment, on the basis of the assigned treatment groups.

**Parameter**	**Overall, *N* = 40**	**Alendronate, *N* =25**	**Denosumab, *N* =15**	***p***
FN-BMD, *N* (%)	23 (57.5)	16 (64.0)	7 (46.7)	0.457
TH-BMD, *N* (%)	25 (62.5)	17 (68.0)	8 (53.3)	0.555
LS-BMD, *N* (%)	29 (72.5)	21 (84.0)	8 (53.3)	0.082
TBS, *N* (%)	15 (37.5)	12 (48.0)	3 (20.0)	0.152
RSMI, *N* (%)	18 (45.0)	10 (40.0)	8 (53.3)	0.622
Hand grip, *N* (%)	18 (45.0)	12 (48.0)	6 (40.0)	0.870

*FN, Femoral neck; LS, Lumbar spine; TH, Total hip; BMD, Bone marrow density; TBS, Trabecular bone score; RSMI, Relative skeletal muscle index*.

Similarly, Figure 2 illustrates the overmentioned longitudinal osteosarcopenic improvements on the basis of the patient's treatment group.

**Figure 3:**
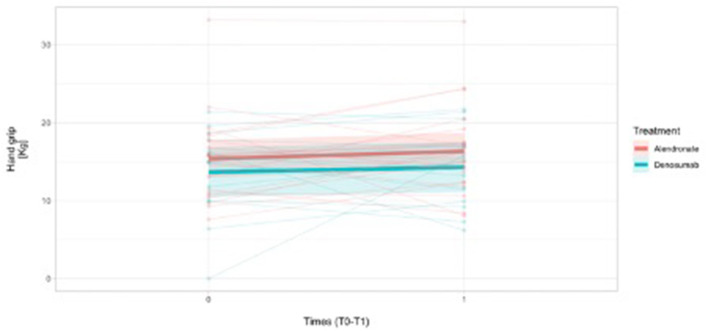
Handgrip measurements trend over time (1 year time-frame observation) in females (*N* = 31) on the basis of the treatment assignment.

In addition, hand-grip longitudinal (T0-T1) measurements were reported in overall 31 female patients and, namely, a mean difference was reported in 19 patients treated with alendronate (19/22) [mean difference To-T1: +0.85 (SD = 4.8) Kg] and in 12/13 females treated with denosumab [mean difference T0-T1: +0.97 (SD = 6.0) Kg], respectively, indicating a positive HG grip trend over time (Figure 3). The missing number of hand-grip measurements were considered as non-responders.

Namely, in males, hand-grip longitudinal measurements(T0-T1) were reported for three patients treated with alendronate (2/3) (+2.3 Kg and +10 Kg from T0) and in one sole patient treated with denosumab (1/2) (−13.3 Kg from T0).

## Discussion

The present findings are preliminary and showed a relatively early trend of OP improvement in both BMD and bone microarchitecture (i.e., TBS) in the alendronate group as compared with denosumab, in a 1-year longitudinal observation.

However, a trend of improvement in sarcopenia (RSMI) was observed in the denosumab group as compared with the alendronate group, and similarly, although the high number of missing data and the prevalent female sex may be a selection bias, the hand-grip measurements showed a positive longitudinal trend in both assignment group.

To the best of our knowledge, although speculative in nature, this is the first report to longitudinally analyze osteosarcopenia in a real-world cohort of very old age patients after hip fracture.

Sparse clinical evidence exists for the impact of osteoporosis treatments in the given old age range, and no clinical trials have exclusively looked at people aged 80 years and more as the primary target.

On one hand, bisphosphonates have been the therapeutic mainstay for decades, and current guidance suggests and initial treatment course for 3–5 years for their early anti-resorptive effect pursuing a net bone density improvement. However, further treatment period might be considered on an individual basis to minimize the risks associated with more prolonged treatment, especially in frail and older populations (38).

On the other hand, it is known that bone histomorphometry findings for denosumab over years 2/3, year 5, and year 10 of treatment are consistent with the mechanism of action of denosumab, which potently inhibits bone resorption and remodeling and increases bone mass and strength over time (39). Namely, denosumab contributes to gains in BMD and may also contribute to reductions in fracture risk by increasing bone matrix strength and stiffness. On the basis of these findings, our limited longitudinal observation (1-year) may count for the unchanged bone density in the denosumab group.

Additionally, persistence of anti-resorptive drugs, including both denousmab and bisphosphonate, is a key factor for the successful management of osteoporosis and fragility fractures, especially in old, multimorbid patients receiving multiple drugs regimens. In the light of the current evidence, a high persistence of denosumab was observed in old-age women with fragility fractures, suggesting the need for further longitudinal analysis on the main determinants of persistence of both anti-resorptive drugs over time in such a comorbid and highly vulnerable population (40, 41).

So far, there is a paucity of data on the role of anti-resorptive drugs on bone microarchitecture in very old individuals, and the present findings seem to support a timely role for alendronate on bone quality compared to denosumab. In contrast with that, denosumab was previously observed to improve TBS independent on BMD in postmenopausal women with osteoporosis (42). However, it is to underscore that TBS assessment at baseline was slightly statistically unbalanced for the regression of the mean effect, creating an overestimation of the 1-year alendronate effect on TBS. Thus, a further statistical adjustment based on a longer clinical trajectories and different time points for osteometabolic assessment is warranted to strengthen this preliminary and partially reliable evidence.

Moreover, in humans, scant data are available on the beneficials effects of denosumab on skeletal muscle function (43), and this is especially true in frail older adults after a highly impacting environmental event, such as hip fracture. In particular, in a proof-of-concept trial, denosumab was reported to improve muscle mass and strength and hand grip in postmenopausal women with osteoporosis for an average duration of 3 years, compared to no treatment. The changes in appendicular lean mass and hand-grip strength were also strongly correlated with changes in lumbar spine BMD.

This scientific background may represent the biological plausibility of our findings and, in particular, the positive longitudinal trend for sarcopenia in the denosumab group may be the platform for further longitudinal assessment and intervention trials in such a highly vulnerable population.

However, the clinical complexity of frail old-age patients, the lack of systematic assessment of clinical, and/or exercise-based intervention targeting osteosarcopenia after hospital discharge and in between the observational period limited the generalization of the findings. Indeed, all patients received postsurgical rehabilitation on the basis of the best clinical practice (44, 45).

However, multiple intervening clinical variables, in between interventional pharmacological and non-pharmacological approaches (e.g., rehabilitation and/or physical therapy) in the time frame observation, may count for substantial clinical instability and heterogeneous frailty trajectories, affecting our ability to understanding the role of both denosumab and alendronate on osteosarcopenia in very old individuals.

In addition, several evidence gaps remain in this area of osteosarcopenia in very old individuals, including a paucity of data on the long-term effects of denosumab or other anti-resorptive agents on matrix mineralization variables, sarcopenia, and clinical outcomes, including any associations between treatment-related changes in bone density, microarchitecture and muscle strength, and long-term fracture outcomes.

Long- and short-term data on the effects of denosumab on bone quality, density, and on sarcopenia variables in very old individuals is warranted. Such a better understanding could help refining the conceptual framework of osteosarcopenia with regard to this highly vulnerable population. In turn, the potential identification of dual anti-resorptive drugs targeting both osteoporosis and sarcopenia is still at its infancy, but if appropriately tested in real-world geriatric trials, it could shed new light on potentially key relevant implications of such dyadic changes on fracture rates and skeletal adverse events over an extended period of uninterrupted denosumab treatment, serving as platforms for therapeutic achievements.

So far, the main limitations of the study are the small sample size and the high discontinuation rate that limited the longitudinal observation and the single population center that may represent a selection bias. In particular, it could be hypothesized that the high drop-put rate of patients relies on their frailty status, including higher posthospital discharge, functional disability, multimorbidity, and poorer social support with increased clinical instability and poorer access to health-care resources and services in the long-term period.

Moreover, there was no randomization and the group assignment was the result of the AIFA regulatory law, that might also count for further assignment biases.

Moreover, we cannot exclude that the improvements of glucose metabolism, neuromuscular function, and overall general health condition and frailty status in old-age patients within the observational period may be at least partially responsible for the improvement in bone and muscle strengths. Second, we did not test for physical performance in order to better understand the severity of sarcopenia and the degree of clinical benefits at the follow-up.

The strengths of the study are the real-world assessment of very old patients after hip fracture and their longitudinal assessment of osteometabolic and sarcopenic parameters for the definition of osteosarcopenia along with the systematic assessment of their clinical phenotypes, based on the comprehensive geriatric assessment. This study is part of an ongoing 3-year longitudinal clinical, and it could be hypothesized that the definition of long-term trajectories along with different time to response and sensitivity analyses for the two treatment groups may add knowledge to the field.

In conclusion, the present preliminary findings, although speculative in nature, moved a step forward in the understanding of “real world” old-age hip fracture patients from a therapeutic standpoint and might help in the identification of the best osteometabolic therapy for long-term treatment, exploring as well the potential dual role of denosumab as an anti-resorptive and muscle-strength-specific drug for osteosarcopenia in this highly vulnerable population.

## Data Availability Statement

The raw data supporting the conclusions of this article will be made available by the authors, without undue reservation.

## Ethics Statement

The studies involving human participants were reviewed and approved by Liguria Regional ethical Commette IRCSS Policlinico san martino, Genoa, Italy. The patients/participants provided their written informed consent to participate in this study.

## Author Contributions

MP, MN, and AC: conceptualization and validation. CG and LP: data analysis and validation. LC: methodology, software, and formal statistical analysis. LM: data curation and validation. GB and AG: investigation and data analysis/interpretation. AN: writing, review, and editing. FS and FM: writing, review, editing, and supervision. All authors contributed to the article and approved the submitted version.

## Conflict of Interest

The authors declare that the research was conducted in the absence of any commercial or financial relationships that could be construed as a potential conflict of interest.
